# Practical Observations on Abortion

**Published:** 1840-10

**Authors:** 


Art. III.—*Practical Observations on Abortion.* By J. S. Streeter, Member of the Royal College of Surgeons, &c. *Plates.—London,* 1840. 8vo, pp. 70.

The author first presented an essay on the subject named in the title-page to the Westminster Medical Society, "on such a wide physiological and pathological basis as he believes can alone lead to judicious measures for the prevention of abortion in future pregnancy, and to the best modes of treatment when the present symptoms proclaim that it must inevitably occur."

"Friends on whose judgment he could rely," advised him to print his essay, and hence the publication before us. The advice was flattering, and compliance followed almost as a matter of course. Mr. Streeter's chief object is, "to direct attention to the impossibility of laying down rules for the prevention of miscarriage in any subsequent pregnancy without first determining from accurate observation, and competent knowledge of embryology, the normal or pathological condition of the aborted ovum, and hereby arriving at the real cause of miscarriage."

We are by no means inclined to regard an enquiry into the conditions of aborted ova as useless or superfluous. The subject is interesting in many points of view, and as at present little more is known than that ova when expelled from the uterus present very various appearances, it is very desirable that they who have the requisite talent and opportunities for entering into such investigations should endeavour to advance our knowledge upon the subject. But we do not perceive how the inspection of an ovum thrown off during one pregnancy can lead us to a knowledge of the best mode of preventing future miscarriage. When women abort several times, the different ova rarely present the same appearances, and therefore a practical guide can seldom be obtained for the prevention of future abortion, by the examination of one of the ova previously expelled. In the majority of cases too we cannot determine whether miscarriage has taken place from a cause operating primarily upon the mother, or upon the ovum itself. We admit exceptions to this general rule; and we admit also that an attentive practitioner may occasionally presume that abortion is threatened from some cause operating upon the mother generally, or upon the uterus especially, and that sometimes too we may suspect the ovum is either degenerated or diseased. But not even with the help of the "wide physiological and pathological basis" of Mr. Streeter, which in sober truth is extremely narrow, can we form any positive opinions upon the subject in the great majority of cases, which can modify our practice. Mr. Streeter's remarks on the treatment of abortion prove the truth of this statement, for they contain nothing more than a very slight, and by no means well-arranged sketch of the mode of treating abortion, which is to be found in every elementary work. We cannot then congratulate Mr. Streeter, at present, upon having advanced our knowledge of the subject of abortion, by his examinations of the "blighted embryonic thing." As he is evidently however an observant and industrious practitioner, we do not despair of his making out a stronger case if he pursue his enquiries.

